# Crystal structure of (1*S*,3*R*,8*R*,9*S*,10*R*)-10-bromo­methyl-2,2-di­chloro-9,10-ep­oxy-3,7,7-tri­methyl­tri­cyclo­[6.4.0.0^1,3^]dodeca­ne

**DOI:** 10.1107/S205698901500657X

**Published:** 2015-04-09

**Authors:** Ahmed Benharref, Lahcen El Ammari, Mohamed Saadi, Moha Berraho

**Affiliations:** a Laboratoire de Chimie des Substances Naturelles, Unité Associé au CNRST (URAC16), Faculté des Sciences Semlalia, BP 2390 Bd My Abdellah, 40000 Marrakech, Morocco; b Laboratoire de Chimie du Solide Appliquée, Faculté des Sciences, Université Mohammed V, Avenue Ibn Battouta, BP 1014, Rabat, Morocco

**Keywords:** crystal structure, β-himachalene derivative, π–π inter­actions

## Abstract

The title compound, C_16_H_23_BrCl_2_O, was synthesized in three steps from β-himachalene (3,5,5,9-tetra­methyl-2,4a,5,6,7,8-hexa­hydro-1*H*-benzo­cyclo­heptene), which was isolated from the essential oil of the Atlas cedar (*cedrus atlantica*). The mol­ecule is built up from two fused six- and seven-membered rings, each linked to a three-membered ring. The six-membered ring has a screw-boat conformation, whereas the seven-membered ring displays a twist-boat conformation. The absolute structure was established unambiguously from anomalous dispersion effects.

## Related literature   

For background to β-himachalene, see: El Haib *et al.* (2011 ▸). For the reactivity of this sesquiterpene and its derivatives, see: El Jamili *et al.* (2002 ▸); Benharref *et al.* (2013 ▸); Zaki *et al.* (2014 ▸). For the synthesis of the title compound, see: Bimoussa *et al.* (2013 ▸). For their potential anti­fungal activity against the phytopathogen *Botrytis cinerea*, see: Daoubi *et al.* (2004 ▸).
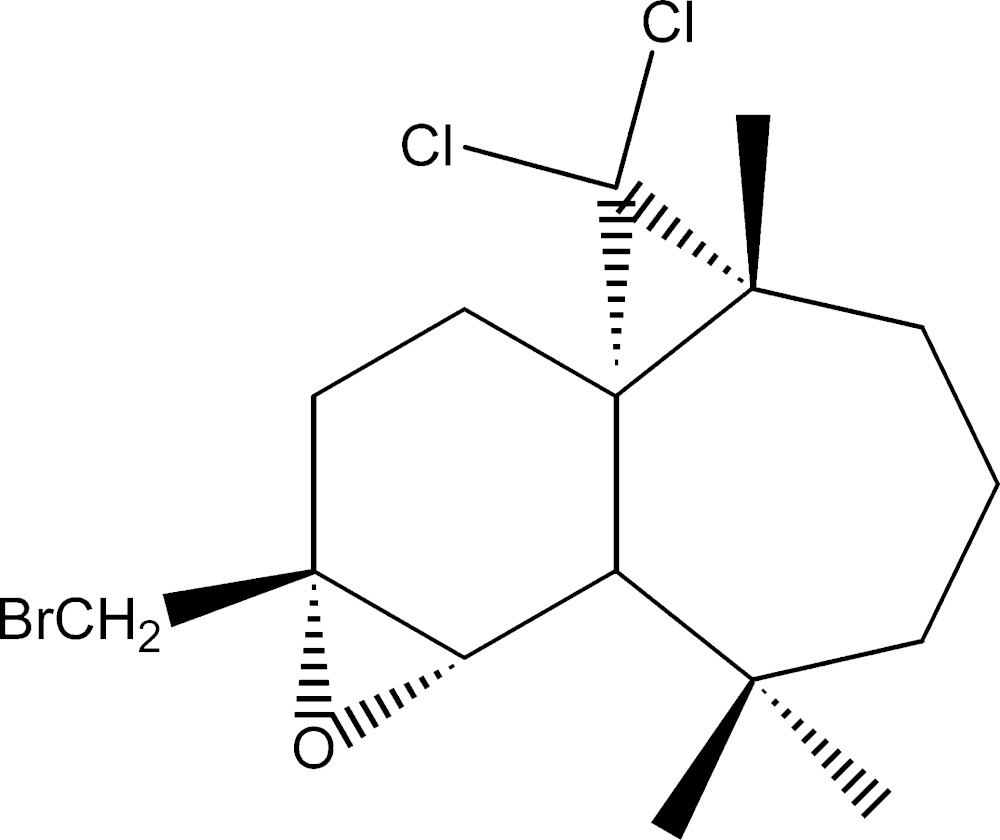


## Experimental   

### Crystal data  

C_16_H_23_BrCl_2_O
*M* *_r_* = 382.15
Orthorhombic, 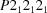

*a* = 8.8748 (5) Å
*b* = 11.2102 (6) Å
*c* = 16.8597 (8) Å
*V* = 1677.34 (15) Å^3^
*Z* = 4
Mo *K*α radiation
μ = 2.76 mm^−1^
*T* = 296 K
0.35 × 0.25 × 0.16 mm

### Data collection  

Bruker APEXII CCD diffractometer
Absorption correction: multi-scan (*SADABS*; Sheldrick, 2003 ▸) *T*_min_ = 0.648, *T*_max_ = 0.746
23384 measured reflections
3419 independent reflections
3135 reflections with *I* > 2σ(*I*)
*R*_int_ = 0.034

### Refinement  

*R*[*F* ^2^ > 2σ(*F* ^2^)] = 0.033
*wR*(*F* ^2^) = 0.088
*S* = 1.04
3419 reflections
184 parameters
H-atom parameters constrained
Δρ_max_ = 0.65 e Å^−3^
Δρ_min_ = −0.53 e Å^−3^
Absolute structure: Flack & Bernardinelli (2000 ▸), 1450 Friedel pairs
Absolute structure parameter: 0.012 (9)

### 

Data collection: *APEX2* (Bruker, 2009 ▸); cell refinement: *SAINT-Plus* (Bruker, 2009 ▸); data reduction: *SAINT-Plus*; program(s) used to solve structure: *SHELXS97* (Sheldrick, 2008 ▸); program(s) used to refine structure: *SHELXL97* (Sheldrick, 2008 ▸); molecular graphics: *ORTEP-3 for Windows* (Farrugia, 2012 ▸); software used to prepare material for publication: *WinGX* (Farrugia, 2012 ▸).

## Supplementary Material

Crystal structure: contains datablock(s) I, global. DOI: 10.1107/S205698901500657X/rz5154sup1.cif

Structure factors: contains datablock(s) I. DOI: 10.1107/S205698901500657X/rz5154Isup2.hkl

Supporting information file. DOI: 10.1107/S205698901500657X/rz5154Isup3.cml

**Fig. 1.**
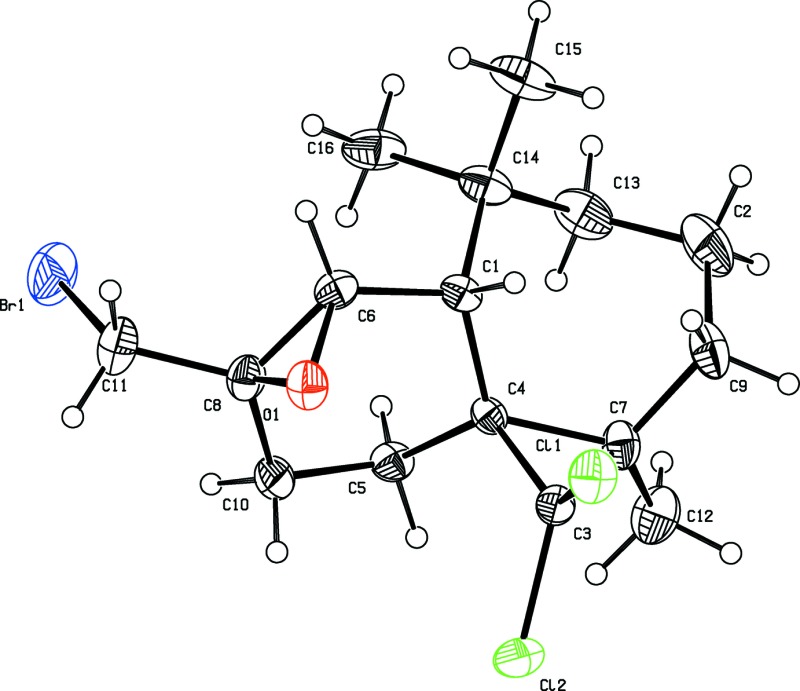
The molecular structure of the title compound with displacement ellipsoids drawn at the 30% probability level. H atoms are represented as small spheres of arbitrary radii.

. DOI: 10.1107/S205698901500657X/rz5154fig1.tif
The mol­ecular structure of the title compound with displacement ellipsoids drawn at the 30% probability level. H atoms are represented as small spheres of arbitrary radii.

CCDC reference: 1057239

Additional supporting information:  crystallographic information; 3D view; checkCIF report

## supplementary crystallographic information

### S1. Comment

This work is a part of our ongoing program concerning the valorization of the most abundant essential oils in Morocco, such as *cedrus atlantica*. This oil is made up mainly (75%) of bicyclic sesquiterpene hydrocarbons, among which is found the compound β-himachalene (El Haib *et al.*, 2011). The reactivity of this sesquiterpene and its derivatives has been studied extensively by our team in order to prepare new products having biological properties (El jamili *et al.*, 2002; Benharref *et al.*, 2013, Zaki *et al.*,2014). Indeed, these compounds were tested, using the food poisoning technique, for their potential antifungal activity against phytopathogen *Botrytis cinerea* (Daoubi *et al.*, 2004). In this work we present the crystal structure of the title compound.

The molecule contains fused six- and seven-membered rings, each linked to a three-membered ring as shown in Fig. 1. The six-membered ring has a screw boat conformation, as indicated by the total puckering amplitude Q_T_ = 0.476 (3) Å and spherical polar angle θ = 129.5 (4)° with φ = 263.4 (5)°, whereas the seven-membered ring displays a twist boat conformation with Q_T_ = 1.1465 (34) Å,θ = 88.54 (18)°, φ2 = -152.04 (18)° and φ3 = 72.86 (6)°. The dihedral angle between the mean planes trough the six- and seven-membered rings is 57.7 (2)°. The three-membered rings (C2/C3/O4) and (C6–C8) are nearly perpendicular to the six-membered ring (C1–C6) with a dihedral angles of 79.8 (3)° and 84.7 (3)°, respectively. Owing to the presence of Cl and Br atoms, the absolute configuration could be fully confirmed, by refining the Flack parameter (Flack & Bernardinelli, 2000) as *S*, *R*, *R*, *S* and *R* for C atoms at positions 1, 3, 8, 9 and 10, respectively.

### S2. Experimental

A stoichiometric quantity of *m*-chloroperbenzoic acid (*m*-CPBA) was added to a 100 ml flask containing a solution of (1*S*,3*R*,8*R*)-10-bromomethyl-2,2-dichloro-3,7,7-trimethyltricyclo[6.4.0.0^1^,^3^]dodec-9-ene (Bimoussa *et al.*, 2013) (750 mg, 2 mmol) in CH_2_Cl_2_ (40 ml). The reaction mixture was stirred at ambient temperature for 2 h, then treated with a 10% solution of sodium hydrogencarbonate. The aqueous phase was extracted with ether and the organic phases were dried and concentrated. Chromatography of the residue on silica (hexane/ethyl acetate, 98:2 *v*/*v*) allowed the isolation of the title compound with a yield of 90% (700 mg, 1.8 mmol). The product was recrystallized from cyclohexane.

### S3. Refinement

All H atoms were fixed geometrically and treated as riding with C—H = 0.96–0.98 Å and with *U*_iso_(H) = 1.2 *U*_eq_(C) or 1.5 *U*_eq_(C) for methyl H atoms.

### Crystal data

**Table:**

C_16_H_23_BrCl_2_O	*F*(000) = 784
*M**_r_* = 382.15	*D*_x_ = 1.513 Mg m^−^^3^
Orthorhombic, *P*2_1_2_1_2_1_	Mo *K*α radiation, λ = 0.71073 Å
Hall symbol: P 2ac 2ab	Cell parameters from 3419 reflections
*a* = 8.8748 (5) Å	θ = 2.2–26.4°
*b* = 11.2102 (6) Å	µ = 2.76 mm^−^^1^
*c* = 16.8597 (8) Å	*T* = 296 K
*V* = 1677.34 (15) Å^3^	Prism, colourless
*Z* = 4	0.35 × 0.25 × 0.16 mm

### Data collection

**Table:**

Bruker APEXII CCD diffractometer	3419 independent reflections
Radiation source: fine-focus sealed tube	3135 reflections with *I* > 2σ(*I*)
Graphite monochromator	*R*_int_ = 0.034
ω and φ scans	θ_max_ = 26.4°, θ_min_ = 2.2°
Absorption correction: multi-scan (*SADABS*; Sheldrick, 2003)	*h* = −11→11
*T*_min_ = 0.648, *T*_max_ = 0.746	*k* = −14→14
23384 measured reflections	*l* = −21→14

### Refinement

**Table:**

Refinement on *F*^2^	Secondary atom site location: difference Fourier map
Least-squares matrix: full	Hydrogen site location: inferred from neighbouring sites
*R*[*F*^2^ > 2σ(*F*^2^)] = 0.033	H-atom parameters constrained
*wR*(*F*^2^) = 0.088	*w* = 1/[σ^2^(*F*_o_^2^) + (0.035*P*)^2^ + 1.0813*P*] where *P* = (*F*_o_^2^ + 2*F*_c_^2^)/3
*S* = 1.04	(Δ/σ)_max_ < 0.001
3419 reflections	Δρ_max_ = 0.65 e Å^−^^3^
184 parameters	Δρ_min_ = −0.52 e Å^−^^3^
0 restraints	Absolute structure: Flack & Bernardinelli (2000), 1450 Friedel pairs
Primary atom site location: structure-invariant direct methods	Absolute structure parameter: 0.012 (9)

### Special details

**Table:**

Geometry. All s.u.'s (except the s.u. in the dihedral angle between two l.s. planes) are estimated using the full covariance matrix. The cell s.u.'s are taken into account individually in the estimation of s.u.'s in distances, angles and torsion angles; correlations between s.u.'s in cell parameters are only used when they are defined by crystal symmetry. An approximate (isotropic) treatment of cell s.u.'s is used for estimating s.u.'s involving l.s. planes.
Refinement. Refinement of *F*^2^ against all reflections. The weighted *R*-factor *wR* and goodness of fit *S* are based on *F*^2^, conventional *R*-factors *R* are based on *F*, with *F* set to zero for negative *F*^2^. The threshold expression of *F*^2^ > 2σ(*F*^2^) is used only for calculating *R*-factors(gt) *etc*. and is not relevant to the choice of reflections for refinement. *R*-factors based on *F*^2^ are statistically about twice as large as those based on *F*, and *R*- factors based on all data will be even larger.

### Fractional atomic coordinates and isotropic or equivalent isotropic displacement parameters (Å^2^)

**Table:**

	*x*	*y*	*z*	*U*_iso_*/*U*_eq_	
C1	0.8156 (3)	0.2763 (2)	0.75464 (16)	0.0311 (5)	
H1	0.8658	0.3365	0.7875	0.037*	
C2	0.7910 (3)	0.3332 (2)	0.67448 (17)	0.0349 (6)	
H2	0.8806	0.3401	0.6408	0.042*	
C3	0.6468 (3)	0.3276 (2)	0.63277 (17)	0.0382 (6)	
C4	0.5134 (3)	0.2638 (3)	0.66859 (18)	0.0439 (7)	
H4A	0.4679	0.2133	0.6285	0.053*	
H4B	0.4389	0.3224	0.6845	0.053*	
C5	0.5541 (3)	0.1874 (3)	0.74026 (17)	0.0362 (6)	
H5A	0.4632	0.1688	0.7697	0.043*	
H5B	0.5976	0.1128	0.7221	0.043*	
C6	0.6652 (3)	0.2497 (2)	0.79482 (15)	0.0286 (5)	
C7	0.6026 (3)	0.3367 (2)	0.85487 (16)	0.0347 (6)	
C8	0.6680 (4)	0.2217 (2)	0.88460 (16)	0.0398 (6)	
C9	0.8207 (4)	0.2258 (3)	0.9237 (2)	0.0570 (9)	
H9A	0.8075	0.2281	0.9808	0.068*	
H9B	0.8719	0.2985	0.9079	0.068*	
C10	0.9190 (5)	0.1185 (4)	0.9020 (3)	0.0755 (13)	
H10A	1.0238	0.1393	0.9107	0.091*	
H10B	0.8946	0.0530	0.9373	0.091*	
C11	0.9007 (5)	0.0760 (3)	0.8162 (3)	0.0603 (10)	
H11A	0.7992	0.0451	0.8104	0.072*	
H11B	0.9693	0.0098	0.8081	0.072*	
C12	0.9274 (3)	0.1671 (3)	0.7491 (2)	0.0457 (7)	
C13	0.5657 (5)	0.1268 (3)	0.9185 (2)	0.0592 (9)	
H13A	0.5498	0.1418	0.9739	0.089*	
H13B	0.6116	0.0499	0.9118	0.089*	
H13C	0.4707	0.1284	0.8913	0.089*	
C14	1.0895 (4)	0.2161 (4)	0.7539 (3)	0.0668 (11)	
H14A	1.1026	0.2583	0.8029	0.100*	
H14B	1.1071	0.2694	0.7103	0.100*	
H14C	1.1597	0.1511	0.7514	0.100*	
C15	0.9119 (4)	0.0994 (3)	0.6701 (3)	0.0586 (10)	
H15A	0.9802	0.0329	0.6697	0.088*	
H15B	0.9354	0.1520	0.6270	0.088*	
H15C	0.8105	0.0709	0.6645	0.088*	
C16	0.6457 (5)	0.3478 (3)	0.54456 (19)	0.0554 (9)	
H16A	0.7275	0.4010	0.5303	0.067*	
H16B	0.5517	0.3854	0.5293	0.067*	
Br1	0.66817 (6)	0.19765 (4)	0.48772 (2)	0.07821 (17)	
O4	0.6911 (3)	0.43512 (16)	0.67456 (12)	0.0418 (5)	
Cl1	0.40574 (9)	0.36000 (8)	0.85958 (5)	0.0521 (2)	
Cl2	0.69214 (10)	0.47409 (6)	0.87514 (5)	0.04980 (19)	

### Atomic displacement parameters (Å^2^)

**Table:**

	*U*^11^	*U*^22^	*U*^33^	*U*^12^	*U*^13^	*U*^23^
C1	0.0274 (12)	0.0251 (11)	0.0408 (14)	−0.0013 (10)	−0.0036 (11)	−0.0025 (10)
C2	0.0353 (13)	0.0311 (13)	0.0383 (14)	−0.0042 (10)	0.0061 (11)	−0.0017 (11)
C3	0.0477 (15)	0.0347 (14)	0.0323 (13)	0.0006 (11)	−0.0016 (12)	−0.0043 (11)
C4	0.0345 (14)	0.0591 (19)	0.0382 (15)	−0.0083 (13)	−0.0069 (12)	−0.0046 (13)
C5	0.0305 (13)	0.0362 (13)	0.0418 (15)	−0.0096 (11)	0.0009 (12)	−0.0039 (12)
C6	0.0271 (11)	0.0265 (11)	0.0323 (12)	−0.0027 (10)	−0.0045 (11)	0.0017 (9)
C7	0.0353 (13)	0.0355 (14)	0.0331 (14)	−0.0013 (11)	−0.0001 (11)	0.0001 (11)
C8	0.0485 (16)	0.0368 (14)	0.0341 (14)	−0.0056 (13)	−0.0056 (13)	0.0077 (11)
C9	0.064 (2)	0.061 (2)	0.0459 (18)	−0.0004 (18)	−0.0205 (18)	0.0138 (15)
C10	0.075 (3)	0.071 (3)	0.081 (3)	0.017 (2)	−0.029 (2)	0.027 (2)
C11	0.0554 (19)	0.0392 (16)	0.086 (3)	0.0153 (15)	−0.009 (2)	0.0085 (17)
C12	0.0330 (15)	0.0369 (15)	0.067 (2)	0.0061 (12)	−0.0024 (14)	−0.0027 (14)
C13	0.076 (3)	0.052 (2)	0.049 (2)	−0.0088 (18)	0.0071 (18)	0.0157 (16)
C14	0.0316 (16)	0.060 (2)	0.109 (3)	0.0024 (15)	−0.0107 (18)	−0.006 (2)
C15	0.0434 (17)	0.0463 (18)	0.086 (3)	0.0076 (14)	0.0107 (18)	−0.0195 (18)
C16	0.079 (2)	0.0550 (19)	0.0326 (15)	0.0012 (18)	−0.0015 (16)	−0.0008 (14)
Br1	0.0966 (3)	0.0883 (3)	0.0497 (2)	0.0133 (3)	−0.0049 (2)	−0.0260 (2)
O4	0.0549 (12)	0.0313 (9)	0.0394 (11)	0.0054 (9)	−0.0052 (10)	0.0000 (8)
Cl1	0.0415 (4)	0.0631 (5)	0.0516 (5)	0.0067 (4)	0.0126 (3)	0.0000 (4)
Cl2	0.0638 (5)	0.0353 (3)	0.0504 (4)	−0.0044 (3)	−0.0037 (4)	−0.0109 (3)

### Geometric parameters (Å, º)

**Table:**

C1—C2	1.510 (4)	C9—C10	1.531 (6)
C1—C6	1.527 (4)	C9—H9A	0.9700
C1—C12	1.579 (4)	C9—H9B	0.9700
C1—H1	0.9800	C10—C11	1.530 (6)
C2—O4	1.446 (3)	C10—H10A	0.9700
C2—C3	1.462 (4)	C10—H10B	0.9700
C2—H2	0.9800	C11—C12	1.543 (5)
C3—O4	1.451 (3)	C11—H11A	0.9700
C3—C16	1.504 (4)	C11—H11B	0.9700
C3—C4	1.509 (4)	C12—C15	1.538 (5)
C4—C5	1.524 (4)	C12—C14	1.542 (4)
C4—H4A	0.9700	C13—H13A	0.9600
C4—H4B	0.9700	C13—H13B	0.9600
C5—C6	1.519 (4)	C13—H13C	0.9600
C5—H5A	0.9700	C14—H14A	0.9600
C5—H5B	0.9700	C14—H14B	0.9600
C6—C7	1.512 (4)	C14—H14C	0.9600
C6—C8	1.546 (4)	C15—H15A	0.9600
C7—C8	1.500 (4)	C15—H15B	0.9600
C7—Cl2	1.766 (3)	C15—H15C	0.9600
C7—Cl1	1.769 (3)	C16—Br1	1.947 (3)
C8—C9	1.507 (5)	C16—H16A	0.9700
C8—C13	1.511 (4)	C16—H16B	0.9700
			
C2—C1—C6	110.7 (2)	C8—C9—H9A	109.1
C2—C1—C12	111.4 (2)	C10—C9—H9A	109.1
C6—C1—C12	115.1 (2)	C8—C9—H9B	109.1
C2—C1—H1	106.3	C10—C9—H9B	109.1
C6—C1—H1	106.3	H9A—C9—H9B	107.8
C12—C1—H1	106.3	C11—C10—C9	114.2 (3)
O4—C2—C3	59.83 (17)	C11—C10—H10A	108.7
O4—C2—C1	114.9 (2)	C9—C10—H10A	108.7
C3—C2—C1	122.6 (2)	C11—C10—H10B	108.7
O4—C2—H2	115.8	C9—C10—H10B	108.7
C3—C2—H2	115.8	H10A—C10—H10B	107.6
C1—C2—H2	115.8	C10—C11—C12	118.1 (3)
O4—C3—C2	59.54 (17)	C10—C11—H11A	107.8
O4—C3—C16	110.9 (2)	C12—C11—H11A	107.8
C2—C3—C16	118.3 (3)	C10—C11—H11B	107.8
O4—C3—C4	114.4 (2)	C12—C11—H11B	107.8
C2—C3—C4	121.0 (2)	H11A—C11—H11B	107.1
C16—C3—C4	117.5 (3)	C15—C12—C14	107.7 (3)
C3—C4—C5	113.4 (2)	C15—C12—C11	107.1 (3)
C3—C4—H4A	108.9	C14—C12—C11	109.9 (3)
C5—C4—H4A	108.9	C15—C12—C1	112.2 (3)
C3—C4—H4B	108.9	C14—C12—C1	107.9 (2)
C5—C4—H4B	108.9	C11—C12—C1	111.9 (3)
H4A—C4—H4B	107.7	C8—C13—H13A	109.5
C6—C5—C4	112.1 (2)	C8—C13—H13B	109.5
C6—C5—H5A	109.2	H13A—C13—H13B	109.5
C4—C5—H5A	109.2	C8—C13—H13C	109.5
C6—C5—H5B	109.2	H13A—C13—H13C	109.5
C4—C5—H5B	109.2	H13B—C13—H13C	109.5
H5A—C5—H5B	107.9	C12—C14—H14A	109.5
C7—C6—C5	117.7 (2)	C12—C14—H14B	109.5
C7—C6—C1	119.5 (2)	H14A—C14—H14B	109.5
C5—C6—C1	112.9 (2)	C12—C14—H14C	109.5
C7—C6—C8	58.76 (18)	H14A—C14—H14C	109.5
C5—C6—C8	120.7 (2)	H14B—C14—H14C	109.5
C1—C6—C8	117.4 (2)	C12—C15—H15A	109.5
C8—C7—C6	61.77 (18)	C12—C15—H15B	109.5
C8—C7—Cl2	120.7 (2)	H15A—C15—H15B	109.5
C6—C7—Cl2	121.82 (19)	C12—C15—H15C	109.5
C8—C7—Cl1	119.6 (2)	H15A—C15—H15C	109.5
C6—C7—Cl1	119.2 (2)	H15B—C15—H15C	109.5
Cl2—C7—Cl1	107.87 (15)	C3—C16—Br1	110.8 (2)
C7—C8—C9	117.9 (2)	C3—C16—H16A	109.5
C7—C8—C13	119.9 (3)	Br1—C16—H16A	109.5
C9—C8—C13	113.3 (3)	C3—C16—H16B	109.5
C7—C8—C6	59.47 (17)	Br1—C16—H16B	109.5
C9—C8—C6	115.9 (3)	H16A—C16—H16B	108.1
C13—C8—C6	120.2 (3)	C2—O4—C3	60.63 (18)
C8—C9—C10	112.6 (3)		
