# Supplementary figures and images for: Crystal structure of (1S,3R,8R,9S,10R)-10-bromo­methyl-2,2-di­chloro-9,10-ep­oxy-3,7,7-tri­methyl­tri­cyclo­[6.4.0.01,3]dodeca­ne
Source: Acta Crystallogr E Crystallogr Commun. 2015 Apr 9;71(Pt 5):o284–5. doi: 10.1107/S205698901500657X (PMC4420093; doi:10.1107/S205698901500657X)

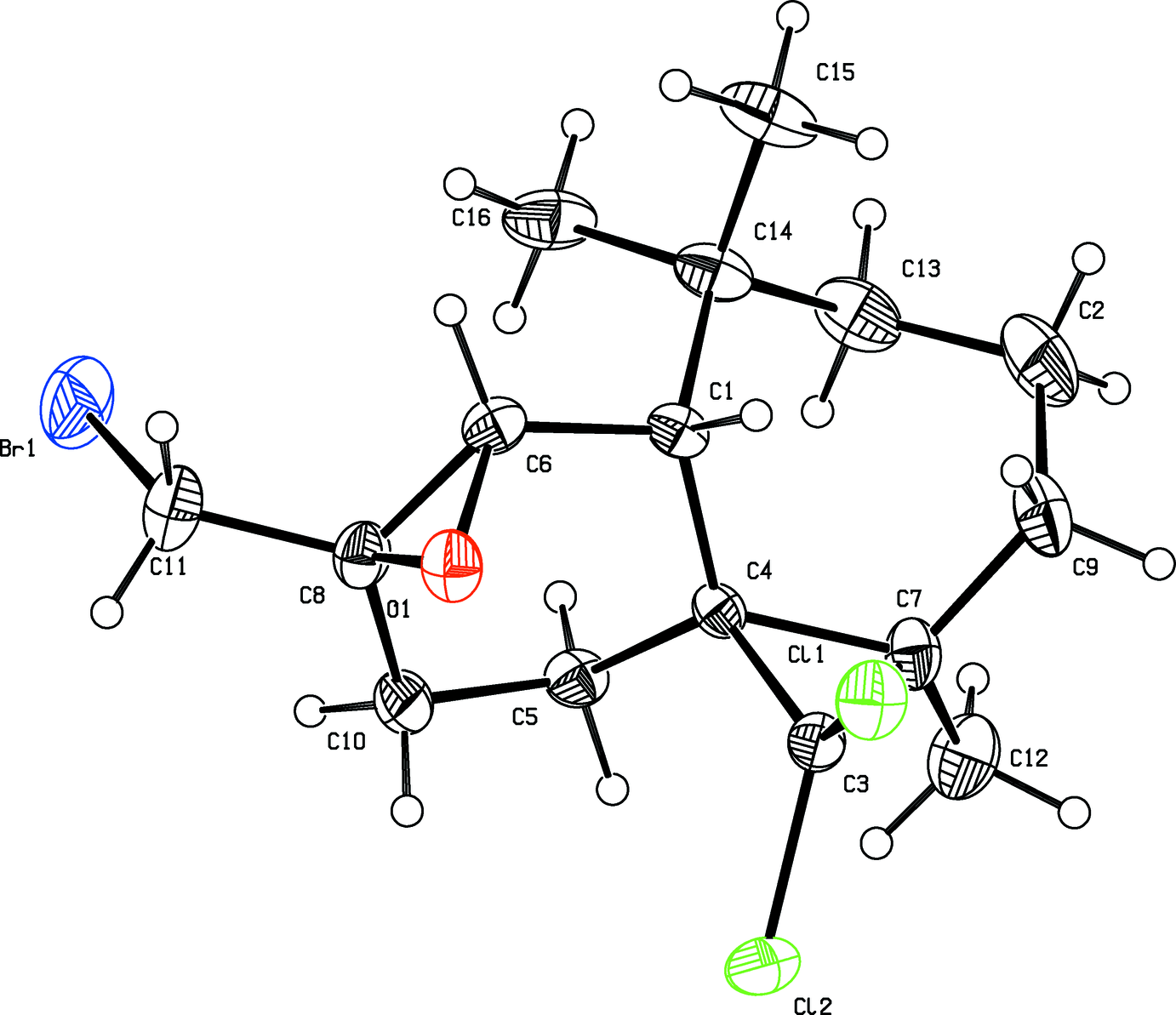

Supplement: Supplementary file 4 [file e-71-0o284-fig1.tif]
